# Height and Depth of the Three Levels of the Face in Second-Trimester Euploid Fetuses

**DOI:** 10.7759/cureus.90129

**Published:** 2025-08-15

**Authors:** Cátia Lourenço, Conceição Brito, Luís Ruano, Ana Vilhena, Mariana Sousa, Vera Afreixo, Horácio Costa

**Affiliations:** 1 Obstetrics, Entre Douro e Vouga Local Health Unit, Santa Maria da Feira, PRT; 2 Department of Obstetrics and Gynecology, Gaia/Espinho Local Health Unit, Vila Nova de Gaia, PRT; 3 Neurology, Entre Douro e Vouga Local Health Unit, Santa Maria da Feira, PRT; 4 Mathematics Department, Aveiro University, Aveiro, PRT; 5 Center for Research and Development in Mathematics and Applications, Department of Mathematics, University of Aveiro, Aveiro, PRT; 6 Plastic and Reconstructive Surgery, Gaia/Espinho Local Health Unit, Vila Nova de Gaia, PRT

**Keywords:** aneuploidy, frontal bone, maxilla, second trimester, sphenoid bone

## Abstract

Objective: To determine sonographic dimensions of the fetal upper, middle and lower facial profile levels in normal pregnancy at the second trimester scan (20-22 weeks).

Methods: This was a prospective, cross-sectional study of 400 normal healthy fetuses at 20-22 weeks of gestation. The sagittal plane of the fetal facial profile was evaluated using transabdominal ultrasound. The three levels of the face were defined as it follows: a vertical line was drawn from the anterior edge of the sphenoid bone and then four parallel lines were drawn parallel to the long axis of the maxilla passing through the inflection point of the frontal bone, the nasion, the superior border of the maxilla and the gnathion.

Results: The study population consisted of 400 low-risk pregnancies at 20-22 weeks gestation. We provided normative data in centiles of height and depth of upper, middle and lower levels of the fetal profile.

Conclusions: We provide normative data of the three levels (upper, middle and lower) of the fetal facial profile between 20-22 weeks. Our data offer a potential tool for the prenatal diagnosis of craniofacial anomalies and a comparative study with trisomy 21 fetuses currently underway.

## Introduction

Cell-free fetal DNA testing is now commonly used to screen for trisomy 21, but ultrasound remains a valuable tool in the prenatal evaluation of genetic and craniofacial abnormalities [1]. Sonographic assessment of the fetal facial profile during the second trimester, typically performed between 20 and 22 weeks of gestation, has become a routine component of the anatomical survey. It plays a key role not only in identifying facial anomalies but also in screening for chromosomal abnormalities, particularly trisomy 21 [2,3].

While three-dimensional (3D) ultrasound can provide detailed images of the fetal face, two-dimensional (2D) ultrasound is generally faster, more accessible, and often sufficient for accurate assessment [2,4]. It is well established that abnormal facial profiles are associated with various syndromic and genetic conditions, many of which may not present with major structural malformations [5].

Nomograms have been developed for several facial structures, including the forehead [6], orbits [7,8], nose [9,10], alveolar ridge [11], mandible [12], philtrum [13,14], and chin [13,15]. However, to our knowledge, no studies have systematically described the proportional measurements - specifically height and depth - of the fetal face in the sagittal plane during the second trimester.

Cossellu et al. introduced the sphenofrontal distance (SFD) as a potential screening marker for trisomy 21 using 3D ultrasound volumes obtained between 16 and 24 weeks of gestation. In their study, 97% of fetuses with trisomy 21 had SFD measurements below the 5th percentile, and 90% were below the 1st percentile, compared to euploid fetuses [16].

The objective of the present study is to establish normative sonographic measurements of the upper, middle, and lower thirds of the fetal facial profile during the second trimester scan (20-22 weeks). These reference values may aid in identifying abnormal facial proportions, which are often associated with facial dysmorphism [3,17].

## Materials and methods

Study design and population

This was a prospective observational study conducted between June 2021 and June 2022 at two Portuguese healthcare institutions: Unidade Local de Saúde (ULS) Gaia/Espinho and ULS Entre Douro e Vouga, EPE. A total of 400 pregnant women were consecutively enrolled. Pregnancy outcomes were followed within the institutions. Neonatal outcome data were unavailable in 32 cases due to deliveries occurring at other institutions and subsequent loss to follow-up.

Inclusion and exclusion criteria

Eligible participants met the following inclusion criteria: 1) Caucasian ethnicity; 2) Singleton pregnancy; 3) Normal findings on first- and second-trimester ultrasounds; 4) Availability of a high-quality mid-sagittal 2D ultrasound image fulfilling the following anatomical criteria: anterior visualization of the nose, upper and lower lips, maxilla, and chin; posterior visualization of the secondary palate, vomeral bone, and corpus callosum; visualization of the anterior portion of the sella turcica, appearing as an echogenic bony structure posterior and superior to the posterior edge of the vomer [1]. The fetal profile was required to occupy at least two-thirds of the image, and all images had to be acquired with the fetal mouth closed.

Ultrasound imaging and data acquisition

Ultrasound examinations were performed at the Prenatal Diagnosis Units of both participating centers by two trained and experienced sonographers. Equipment used was the GE Voluson E8 (GE Healthcare, Chicago, IL, USA). All images were acquired transabdominally, exported in Digital Imaging and Communications in Medicine (DICOM) format, and subsequently analyzed using Astraia software (Ismaning, Germany). Fetal facial landmarks were measured with RadiAnt DICOM Viewer (Medixant, Poznań, Poland).

Ethical considerations

This study was approved by the local Ethics Committees of both participating institutions. All procedures adhered to the principles outlined in the Declaration of Helsinki.

Measurement protocol

**Figure 1:**
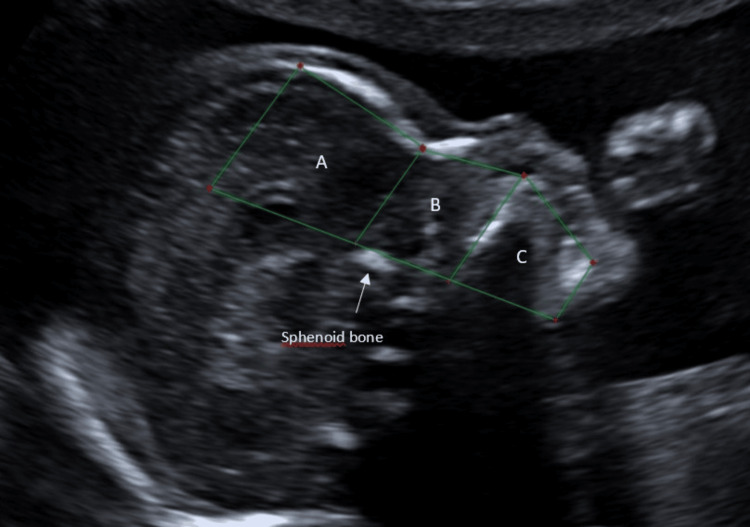
Measurements of height and depth of the three levels of the face in a euploid fetus A: superior level of the face B: middle level of the face C: inferior level of the face

Fetal facial measurements were obtained based on a segmentation of the facial profile into three anatomical levels: upper, middle, and lower thirds. The method consisted of the following steps: 1) A vertical reference line was drawn through the anterior border of the sphenoid bone; 2) A baseline was defined parallel to the long axis of the maxilla; 3) Additional parallel lines to the long axis of the maxilla were drawn passing through three anterior landmarks: the inflection point of the frontal bone, the nasion, and the gnathion (Figure 1). These lines demarcated the facial profile into the following three levels: upper, middle and lower levels.

The boundaries of the upper level are as follows: the posterior limit is a vertical line through the anterior border of the sphenoid bone; the anterior limit is a line connecting the leading edge of the frontal bone to the nasion; the superior limit lies from the frontal bone (parallel to the maxilla) to the vertical sphenoid line and the inferior limit lies between the nasion (parallel to the maxilla) to the vertical sphenoid line.

Regarding the middle level, the posterior limit is a vertical line through the anterior border of the sphenoid bone; the anterior limit is a line from the nasion to the most anterior point of the maxilla; the superior limit goes from the nasion (parallel to the maxilla) to the sphenoid line and the inferior limit is the maxilla’s long axis.

Concerning the lower level, the posterior limit is a vertical line through the anterior border of the sphenoid bone; the anterior limit is a line between the maxilla and the gnathion; the superior limit is the long axis of the maxilla and the inferior limit goes from the gnathion (parallel to the maxilla) to the vertical sphenoid line.

The nasion was defined as the most anterior point at the intersection of the frontal and nasal bones [2].

## Results

The study population consisted of 400 singleton pregnancies. The median maternal age was 31 years (18-49 years), and the median body mass index (BMI) was 24 kg/m² (16.5-44.5 kg/m²). Gestational age at the time of ultrasound evaluation ranged from 20 to 22 weeks. The median gestational age at delivery was 39 weeks (24.0-41.6 weeks), and the median neonatal birthweight was 3175 grams.

Reference ranges (expressed in percentiles) were established for the depth and height of the three anatomical segments of the fetal face: upper, middle, and lower thirds.

For the upper third of the fetal face, the following values were recorded at the 50th percentile: superior limit: 16.1 mm ( 11.5-22.6 mm); inferior limit: 14.5 mm (13.0-18.2 mm); anterior limit: 15.4 mm (12.4-19.0 mm); posterior limit: 15.9 mm (12.4-19.1 mm) (Table 1).

**Table 1:** Percentile distribution of dimensions of anterior, posterior, superior and inferior limits of upper level of the face

Centile (th)	Upper level – superior limit	Upper level – inferior limit	Upper level – anterior limit	Upper level – posterior limit
5	11.5	13.0	12.4	12.4
10	11.9	13.4	13.5	13.3
15	12.5	13.4	13.9	14.0
20	13.9	13.6	14.4	14.3
25	14.2	13.7	14.6	14.6
30	14.5	14.0	14.8	14.9
35	15.3	14.0	14.9	15.0
40	15.5	14.1	15.1	15.1
45	15.8	14.3	15.2	15.4
50	16.1	14.5	15.4	15.9
55	16.3	14.8	16.0	16.0
60	16.5	15.3	16.9	16.6
65	17.0	15.4	17.1	17.0
70	17.1	15.8	17.4	17.2
75	17.7	16.0	17.4	18.0
80	18.2	16.2	17.5	18.1
85	18.9	16.4	17.9	18.2
90	20.6	16.8	18.4	18.6
95	22.6	18.2	19.0	19.1

For the middle third of the fetal face, the 50th percentile measurements were: superior limit: 14.5 mm (13.0-18.2 mm); inferior limit: 18.5 mm (14.6-21.9 mm); anterior limit: 14.2 mm (11.6-16.8 mm); posterior limit: 13.1 mm (10.8-15.7 mm) (Table 2).

**Table 2:** Percentile distribution of dimensions of anterior, posterior, superior and inferior limits of middle level of the face

Centile (th)	Middle level – superior limit	Middle level – inferior limit	Middle level – anterior limit	Middle level – posterior limit
5	13.0	14.6	11.6	10.8
10	13.4	15.0	12.2	11.1
15	13.4	16.3	12.5	11.5
20	13.6	16.9	12.7	11.7
25	13.7	17.6	12.9	11.9
30	14.0	17.8	13.5	12.1
35	14.0	17.8	13.7	12.6
40	14.1	17.9	13.8	12.7
45	14.3	18.4	13.9	12.9
50	14.5	18.5	14.2	13.1
55	14.8	18.7	14.4	13.3
60	15.3	18.8	14.5	13.4
65	15.4	18.8	14.7	13.7
70	15.8	19.2	15.1	13.9
75	16.0	19.4	15.2	14.3
80	16.2	20.0	15.4	14.5
85	16.4	20.3	16.1	14.7
90	16.8	20.7	16.6	15.1
95	18.2	21.9	16.8	15.7

In the lower third of the fetal face, the 50th percentile values were: superior limit: 18.5 mm (14.6-21.6 mm); inferior limit: 12.8 mm (8.7-19.3 mm); anterior limit: 13.3 mm (10.4-16.5 mm); posterior limit: 13.4 mm (range: 10.7-16.8 mm) (Table 3).

**Table 3:** Percentile distribution of dimensions of anterior, posterior, superior and inferior limits of lower level of the face

Centile (th)	Lower level – superior limit	Lower level– inferior limit	Lower level – anterior limit	Lower level – posterior limit
5	14.6	8.7	10.4	10.7
10	15.0	9.3	11.2	11.0
15	16.3	9.7	11.9	11.5
20	16.9	10.1	12.1	12.3
25	17.7	11.0	12.6	12.6
30	17.8	11.4	12.8	12.7
35	17.8	11.8	12.9	12.7
40	17.9	12.1	13.0	13.0
45	18.4	12.5	13.2	13.2
50	18.5	12.8	13.3	13.4
55	18.7	13.3	14.0	13.5
60	18.8	13.6	14.4	13.7
65	18.8	14.0	14.6	13.9
70	19.0	14.2	14.8	14.2
75	19.4	14.4	14.8	14.4
80	20.0	15.1	15.1	14.4
85	20.3	15.4	15.2	14.9
90	20.7	16.9	16.0	15.3
95	21.6	19.3	16.5	16.8

Intraobserver agreement, assessed using the intraclass correlation coefficient (ICC), ranged from 0.775 to 0.921 across all facial measurements, indicating good to excellent reliability. Interobserver ICC values ranged from 0.795 to 0.850 for most parameters, except for the anterior and posterior limits of the middle facial segment, which showed slightly lower values of 0.738 and 0.707, respectively.

These findings demonstrate good reproducibility and reliability of the measurement methodology applied in this study.

## Discussion

In our study, we established reference values for the normal dimensions and proportions of the fetal facial profile at 20-22 weeks of gestation in pregnancies with normal first- and second-trimester ultrasounds. The acquisition of these measurements is feasible and can be easily incorporated into routine second-trimester ultrasound evaluations as fetal profile assessment is already standard practice for the evaluation of the nasal bone and corpus callosum [18].

It is anticipated that deviations from these normative values may facilitate the prenatal identification of craniofacial abnormalities. Nevertheless, in order to determine the clinical utility and diagnostic performance of these parameters, larger prospective studies are needed to evaluate their sensitivity and specificity in detecting both isolated and syndromic craniofacial anomalies, including those associated with aneuploidy [17].

In particular, comparing the reference measurements established in our study with those obtained from fetuses with known craniofacial anomalies, such as trisomy 21, could provide valuable insights. Fetuses with trisomy 21 typically present with midface hypoplasia. Alio et al. reported a reduced distance between the sella turcica and the nasion in children and adolescents with trisomy 21 [18]. Similarly, Cossellu et al. found that the SFD was below the 5th centile in over 90% of fetuses with trisomy 21 [16]. In these cases, the sphenoid bone appears closer to the frontal bone and is located more cranially than in euploid fetuses. Additionally, the shape of the sphenoid bone itself may differ; for example, Korayem and Alkofide observed that in trisomy 21 patients, the anterior wall of the sphenoid bone was more frequently oblique, a feature that may manifest prenatally as increased irregularity [19]. This alteration could result in a shorter measured SFD, depending on the anatomical landmarks chosen-particularly if only the most anterior point of the sphenoid is used as a reference [1].

Postnatal imaging studies have confirmed that individuals with trisomy 21 exhibit reduced growth of the midface, frontal bone, cranial base, and sagittal portion of the endocranium. These anatomical variations contribute to vertical hypoplasia of the central cranial structures, displacement of the sella turcica, and flattening of the cranial base [16]. In contrast to findings by Abele [1], other studies consistently show that the anterior cranial base is significantly shorter in trisomy 21 patients compared to normal controls [16]. The findings of Cossellu et al. suggest that part of the characteristic flat facial appearance in trisomy 21 may be attributable to a shortened sphenofrontal distance, potentially resulting from posterior displacement of the lower frontal bone [16]. Based on this evidence, we would expect measurable differences, particularly in the upper and middle facial segments, when comparing euploid fetuses to those with trisomy 21, thereby potentially validating the clinical relevance of our results.

While current trends in prenatal screening emphasize first-trimester ultrasound assessments and cell-free DNA testing, a significant proportion of the global pregnant population still lacks access to these technologies. For these women, comprehensive second-trimester ultrasound remains critical. In this context, the evaluation of fetal facial morphology may serve as a valuable adjunct tool in the early detection of structural anomalies and a broader range of genetic syndromes beyond the common trisomies [2].

The present study characterizes the distribution of measurements of the three levels of the face in euploid fetuses. Comparing these baseline data with measurements from fetuses exhibiting craniofacial anomalies, such as trisomy 21, is expected to yield valuable insights into the morphological distinctions associated with these conditions. Such comparative analyses are currently underway as part of an ongoing investigation by our group.

## Conclusions

We established normative values for the height and depth of the three facial levels in fetuses at 20-22 weeks of gestation, with high interobserver and intraobserver reproducibility. A forthcoming comparative study with fetuses with trisomy 21 will further clarify the clinical value of these measurements in prenatal screening and diagnosis.
